# Numerical study on the evolution process of slope failure triggered by extreme rainfall along a road-cut in mountainous terrain

**DOI:** 10.1038/s41598-022-10655-5

**Published:** 2022-04-26

**Authors:** Fhatuwani Sengani, Dhiren Allopi

**Affiliations:** 1 Department of Civil Engineering and Geomatics, Faculty of Engineering and the Built Environment, Durban University of Technology, P. O. Box 1334, Durban, 4000 South Africa; 2 Department of Geology and Mining, University of Limpopo, Private Bag X1106, Sovenga, 0727 South Africa

**Keywords:** Civil engineering, Natural hazards

## Abstract

Modeling the flow evolution of a slope governed by solid mass has been recognized as a challenge, yet most stability analyses are only based on stability number or Safety Factor (FOS). The stability number in most cases does not incorporate the deformation characteristics of the material and the change in solid mass phases such as from solid-like to fluid-like phase. Therefore, the purpose of this study is to present a numerical simulation that describes the failure evolution of a slope with a fault along with a road cut. A finite element method associated with an elastoplastic model with strain softening is adopted to provide a failure evolution of R71 road cut slope instabilities. The results of the study have demonstrated that the present computational framework is capable of quantitatively reproducing the failure evolution process, the final run-out distance of the slope material. The simulation has evidenced that the flow evolution of material during extreme rainfall is expected to extend to the final deposit of 4.5 m, indeed, the field measurements and observations also confirm. Furthermore, the simulations also demonstrated that the distance in which material can reach is largely controlled by the composition and phases of the material undergone during flow evolution. Owing to that, the resistance of material has a major role in the run-out of the material; this resistance of the material is also controlled by shearing and absorbed kinetic energy during the process. The overall conclusion is that, for material to flow for a longer distance, high kinetic energy and more shearing of material are expected to take place during this process. It is recommended that other sophisticated methods could be utilized to further the results.

## Introduction

Rockfalls are the most common type of slope movement that makes rock cuts along transportation corridors in mountainous regions hazardous^1^. It is essential to understand the deformation characteristics and failure mechanism driving the instability of slopes^2,3^. Slope stability refers to the potential of the earth’s surface material (soil and rocks) on inclined slopes to withstand or undergo movement. The strength and cohesion of the slope material as well as the amount of internal friction between materials help in maintaining the stability of the slope. Stability is determined by the angle of the slope and the strength of the constitutive material. The steepest angle that a cohesionless slope can maintain the mass without losing its stability is referred to as its angle of repose. Slope stability also refers to the relationship between the driving and the resisting forces. The ratio of resisting forces to driving forces is known as the factor of safety of the slope. The gravitational force is evidently the main driving force acting on a slope. The gravity-induced driving force is directly proportional to the slope inclination. The existence of discontinuities in the rock leads to uneven distribution of strength and stress in all directions. Elastic properties of the rock mass are consequently altered leading to the disrupted balance of rock mass strength as well as landslides. The orientation of discontinuities is another major factor affecting rock stability and rock failure alike^3^.

Rock slope failures can be classified depending on the type and degree of structural control. The most encountered rock slope failures include planar, wedge, toppling, and circular failures. The geometry of the slope, the characteristics of the potential planes of failure, surface drainage, and groundwater conditions are the main internal factors controlling rock slope stability. Rainfall, seismicity, and man-made activities, on the other hand, are external factors. The combination of these factors are responsible for the conditions of stability of a slope^4^.

The analysis of rock slope stability is generally performed to assess how safe and functional excavated and natural slopes are. Eberdhart^5^ argues that the analysis is aimed at assessing slope conditions, potential failure mechanisms, and slope response to external factors that can trigger failure. Moreover, the analysis can be used not only to determine the most effective options for support stabilization but also to optimally design safe, reliable and economical excavation slopes. This analysis mostly revolves around the concept of the factor of safety.

The Factor of Safety (FoS) can be defined in three different ways: limit equilibrium, force equilibrium and moment equilibrium^6^. It should be noted that the factor of safety of a rock slope is usually assessed through a detailed comparison of the calculated FOS against the acceptable FoS. Hoek^7^ argued that the acceptable standard threshold value of FOS is 1.5 for road rock slopes before failure occurs.

Limit equilibrium methods are known to suffer from the uncertainty associated with estimated input parameters amongst others. To overcome this, probability methods are usually resorted to^8^. Most of these methods replace FOS values with a probability of failure as a measure of slope stability (see^9–16^. Yet most of the landslides, rockfalls, and slope stability analysis centered on limit equilibrium methods.

The analysis of the stability of rock slopes is performed primarily to assess their safety^17–20^, the selection of the analysis tool depends on the conditions of the site and its expected mode of failure^21,22^. The challenge has been in finding adequate tools that can describe failure as a nonlinear phenomenon involving solid-like and liquid-like behaviors. The latter known as Fluid-Solid-Structure Interaction (FSSI) cannot be solved using, for example, the classical Arbitrary Lagrangian–Eulerian (ALE) formulation of the FEM framework^23–25^. However, most of these methods suffer to provide flow evolution of the material, yet it is always voiced on how the slope instability occurred.

Over the last 2 decades, a framework known as the Particle Finite Element Method (or PFEM) has been explored in the field of geotechnical engineering. The numerical modelling paradigm is a combination of finite element and meshless finite element methods. It revolves around the numerical resolution of the Lagrangian formulation of particulate systems. The technique has gained popularity thanks to pioneering work by Aubry et al.^24^, Idelsohn et al.^25,26^, Onate et al.^27,28^, and Larese et al.^29^. Originally, the PFEM was intended to solve solid particulate systems interacting with fluid^30^. It is widely used in fluid mechanics because it allows for mesh distortion while following the evolution of free surfaces^30–36^. The PFEM has been well established in solving large deformation problems, yet shallow deformation problems remain unsolved or still not well understood. Therefore the aim of this study is to conduct a numerical simulation on the evolution process of slope failure triggered by extreme rainfall along a road-cut in mountainous terrain. The scope of this study is limited to the determination of the final run-out distance of the material and also determination of shear dissipation of the material when material roll from the top to the bottom part of the slope. Owing to that a new framework called Optimum G2 was applied to strive to establish the flow evolution of this slope. The Optimum G2 is a 2D model with the capability of simulating the flow evolution of the material (rockfall and soil sliding) based on it computational cycles. Indeed, the road cut slope along the R71 was selected as a case study. This road had been reported to experience slope instability of different kids such as rockfall and soil sliding, however, the focus in this study is rockfall which is influence by a fault and it is believed that the initial of rock failure is associated with extreme rainfall, since most of this events are reported during or after heavy rainfalls.

The study commence with an introductory section which has provided the general background of the study and aim, followed by the locality of the study area, methodology, results and discussion and then conclusions.

## Background of the study area

**Figure 1:**
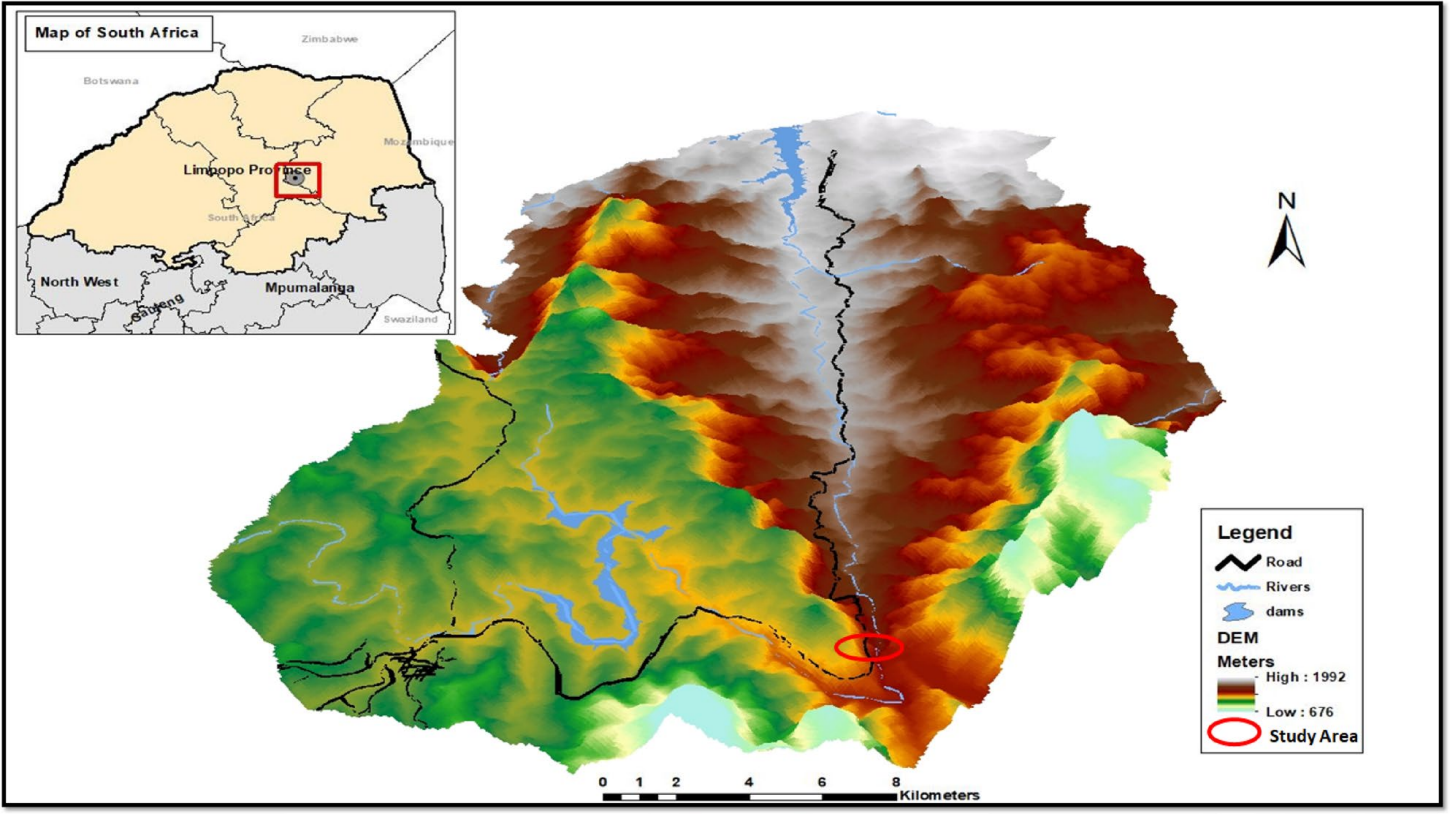
Location of Regional Road (R71) networks. Note that the map was developed using DEM tool in ArcGIS 10.2.2, the software package is available at the institution.

**Figure 2:**
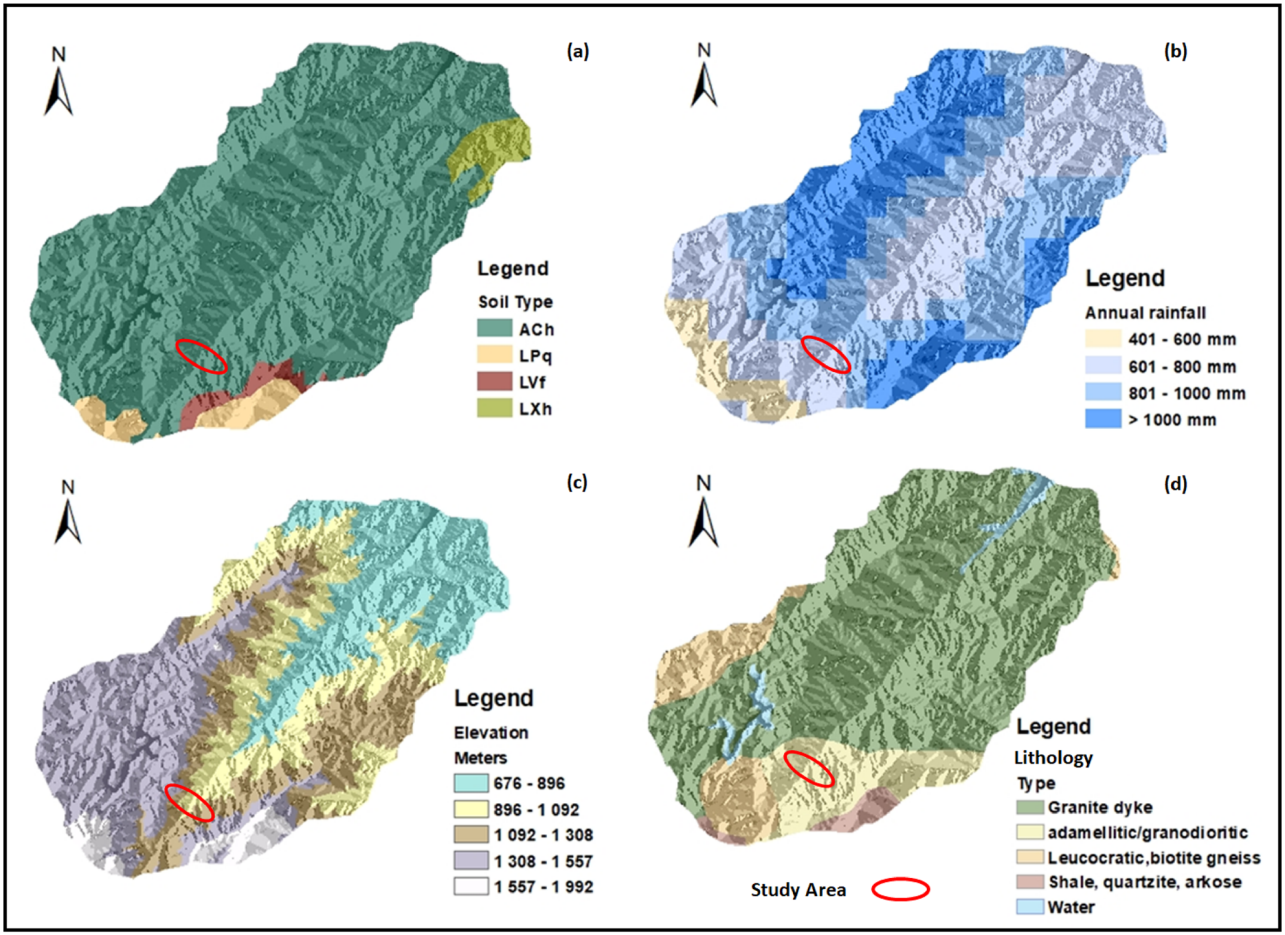
Environmental and geological conditions of the study (**a**) soil type (ACH = silt clay loam, LPq = Loam sand, LVF = clay loam, LXh = silt clay); (**b**) annual rainfall; (**c**) elevations; (**d**) lithology. Note that maps were developed using DEM tool in ArcGIS 10.2.2, the software package is available at the institution.

The study was conducted at Regional Road (R71) located in a Steep and Mountainous Terrain of Limpopo Province, South Africa (Fig. 1). R71 connects Polokwane with Kruger national park through major towns such as Tzaneen and Phalabora. The regional road can be found along the two coordinate points starting from 23° 53′ 54.7″ S, 29° 30′ 39.9″ E and ending at 23° 56′ 44.5″ S, 31° 09′ 54.0″ E. Although the length of the road extent to 201 km from East to West of Limpopo Province, this study is going to focus mostly on the Steep and Mountainous Terrain of Makgoebaskloof mountains (23° 56′ 53.3″ S, 29° 57′ 11.1″ E and 23° 54′ 32.7″ S, 30° 03′ 45.2″ E). The locality map was developed using DEM tool in ArcGIS 10.2.2, similarly to the Fig. 2, same software was utilized to develop the maps.

The study area contain various rock types, which include granites, gneiss, shales, quartzite and sandstone. Nevertheless, a large portion of the area is dominated by granites. Furthermore, there are several soil types as shown in Fig. 2a, the soil types various from sandy soil to clay soil. Owing to that, the annual rainfall of the study area is also documented in Fig. 2b, while lithology and elevation are shown in Fig. 2c,d, respectively.

As already stated the R71 has been well known to experience slope instabilities along the road cut. This problem has been there for several decades, yet the mechanism behind this failure is still not clear, similarly the flow evolution of the failed sloped is still not understood, yet the present short technical note on striving to provide an explanation on failure evolution has been suggested.

## Methodology of the study

The methodology of the study incorporates the governing equation of the framework used and the numerical procedure of the model. The detailed description of the methodology is discussed below.

### Field observations and measurements

The data collection was performed through field observations and measurements. Here, geological rock units’ information and field measurements of the rock mass structures were conducted within the selected study areas. Observations were focusing on the rock conditions as well as evidence of slope instability across the selected study areas. Indeed, field measurements were more focused on collecting the rock mass properties as shown in Table 1. In terms of observations, the rock mass of interest in this study was governed by Granodiorite, with very limited joint sets and a fault cutting across the rock unit. Nevertheless, the fault was observed to present very limited infill materiel, in fact the fault had no infill material. It was also observed that the rockfall occurred across the selected slope occurred along the fault plane and as such broken rock unit of Granodiorite were observed at the bottom part of the slope. Majority of the broken rock are in boulders and some of the blocks are still intact (see Table 1). Yet the understanding on how the failure occurred is significant.

**Table 1:** Material properties of the selected slope.

	Parameters			Parameters		Overview of the Slope studied
Rock mass classification	Sigci (MPa)	250	Failure envelope range	Application	Slopes	Image A Image B Shear and normal stress plot of rock mass
	GSI	75		sig3max (MPa)	0.312268	
	Mi	29		Unit weight (MN/m^3^)	0.026	
	D	1		Slope height (m)	10	
Hoek Brown criterion	Mb	4.86264	Rock mass parameters	sigt (MPa)	−0.79709	
	S	0.0155039		sigc (MPa)	31.0107	
	A	0.500911		sigcm (MPa)	76.5769	
Mohr–Coulomb fit	C (MPa)	2.93988		Em (MPa)	21,084.8	
	Phi (degrees)	68.3739	Fault properties	Density (kg/m^3^)	2500.0	
General properties of rock slope	Dip (°)	65^o^		Cohesion (Pa)	100,000.0	
	Dip direction (°)	N 130° E		Tension (Pa)	0	
	Height, (m)	7		Friction angle (°)	18	
	Nature of slope	Man-made slope		Dilation angle (°)	0	
	Geology	A granodiorite with few joint sets cutting across the rock mass with minor weathering and a fault cutting cross (see Image A). A granite dyke, extremely weathered and blocky few several joint sets (see Image B). Note the interest is on the granodiorite slope failure				

**Figure 3:**
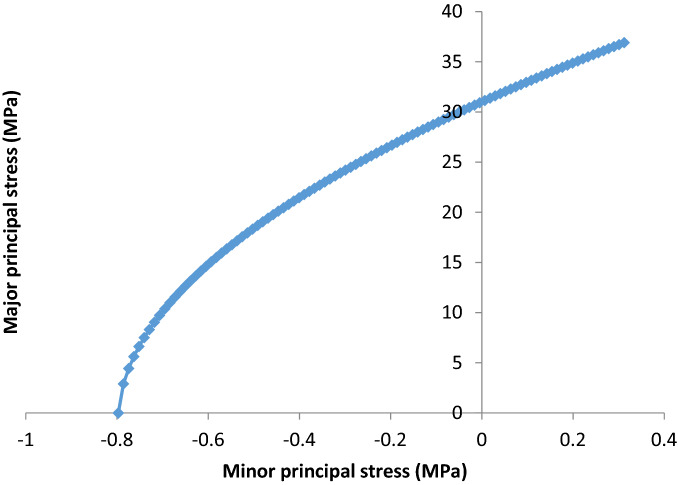
Plot on the relationship between major and minor principal stress of the rock mass provided there is change in stress with time.

**Figure 4:**
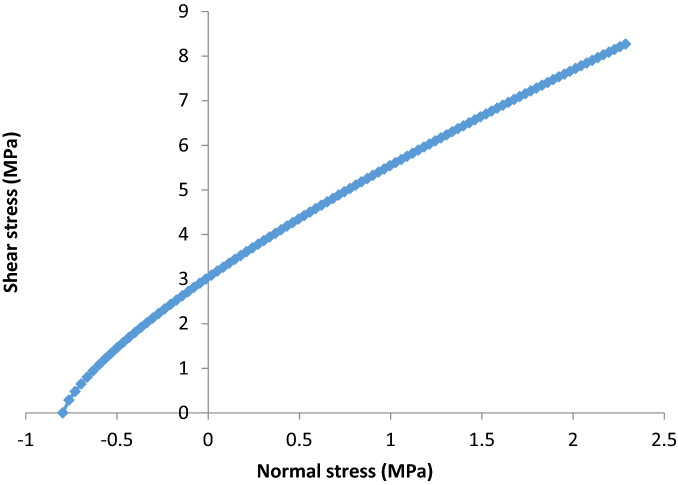
Plot on the relationship between shear and normal stress of the rock mass provided there is change in stress with time.

Nevertheless, the observed and measured rock properties were therefore applied in a Roclab model to estimate the rock mass classification, Hoek and Brown criterion properties, Mohr–Coulomb fit, rock mass properties, failure envelope range of rock mass, fault properties and general properties of rock slope. In terms of rock mass classifications, the model was able to estimate Uniaxial compressive strength of the rock (sigci), Geological Strength Index and contact value of rock mass (mi) as well as the disturbance rate (D). The so called Geological Strength Index (GSI) was used to predict both rockmass and fault strength. The GSI criterion was introduced by Hoek^39,40^, Hoek, et al.^41^, and Hoek et al.^42^ provided a number which, when combined with the intact rock properties, can be used for estimating the reduction in rock mass strength for different geological conditions. The GSI has been extended to cater for blocky rock masses, heterogeneous rock masses such as flysch and Molassic rocks^43^ and Ophiolites^44^. Similarly, the degree of disturbance (D factor) on the rockmass and the uniaxial compressive strength of the rockmass has been estimated based on developed charts by Hoek and Brown^45^.Similarly to rock mass classification other parameters were also identified as shown in Table 1. Furthermore, the model also simulated the relationship between major and minor stress acting on the rock mass, provided there is an increase in stress with time, following the a relationship between shear stress and normal stress was also simulated by the model (see Figs. 3, 4).

### Governing equations

Governing equations defining the mathematical implementation of the FEM model of a solid system are encapsulated in Eqs. (1–9). Assuming an infinitesimal deformation is to be simulated, the equations can be expressed as follows^37^.

1 $$ \varepsilon = \nabla u $$

where $$u={\left[\begin{array}{ccc}{u}_{x}& {u}_{y}& {u}_{z}\end{array}\right]}^{T}$$ are the displacements while the strains $\varepsilon$ are given by

2 $$ \varepsilon = \left[ \begin{gathered} \, \varepsilon_{x} \hfill \\ \, \varepsilon_{y} \hfill \\ \, \varepsilon_{z} \hfill \\ 2 \, \varepsilon_{xy} \hfill \\ 2 \, \varepsilon_{yz} \hfill \\ 2 \, \varepsilon_{zx} \hfill \\ \end{gathered} \right] = \left[ \begin{gathered} \, \frac{{\partial u_{x} }}{\partial x} \hfill \\ \, \frac{{\partial u_{y} }}{\partial y} \hfill \\ \, \frac{{\partial u_{z} }}{\partial z} \hfill \\ \frac{{\partial u_{x} }}{\partial y} + \frac{{\partial u_{y} }}{\partial x} \hfill \\ \frac{{\partial u_{y} }}{\partial z} + \frac{{\partial u_{z} }}{\partial y} \hfill \\ \frac{{\partial u_{z} }}{\partial x} + \frac{{\partial u_{x} }}{\partial z} \hfill \\ \end{gathered} \right] $$

In Eqs. (2.12), the term $\nabla$ is the usual linear strain–displacement operator taking the following form

3 $$ \nabla = \left[ \begin{gathered} \begin{array}{*{20}c} {\frac{\partial }{\partial x}} & 0 & 0 \\ 0 & {\frac{\partial }{\partial y}} & 0 \\ 0 & 0 & {\frac{\partial }{\partial z}} \\ \end{array} \hfill \\ \begin{array}{*{20}c} {\frac{\partial }{\partial x}} & {\frac{\partial }{\partial y}} & 0 \\ 0 & {\frac{\partial }{\partial y}} & {\frac{\partial }{\partial z}} \\ {\frac{\partial }{\partial x}} & 0 & {\frac{\partial }{\partial z}} \\ \end{array} \hfill \\ \end{gathered} \right] $$

The general three-dimensional differential equations of equilibrium are given by.

Equilibrium and static boundary conditions of the model:

4 $${\nabla }^{T}\sigma +b=0,\mathrm{ in V}$$

where $$\sigma ={\left[\begin{array}{ccc}{\sigma }_{x}& {\sigma }_{y}& {\sigma }_{z}\end{array} \begin{array}{ccc}{\sigma }_{xy}& {\sigma }_{yz}& {\sigma }_{zx}\end{array}\right]}^{T}$$ are the stresses, $$b={\left[\begin{array}{ccc}{b}_{x}& {b}_{y}& {b}_{z}\end{array}\right]}^{T}$$ are the body forces stemming for example from self-weight, and V is the domain under consideration.

5 $${\mathrm{P}}^{T}\sigma =\alpha t \mathrm{on }{S}_{\sigma }$$

Meanwhile, the yield conditions of the model are as follows:

6 $${F}^{T}\sigma -k+s=0$$

Since the current situation is dealing with strain problems, therefore associate flow rules or strain–displacement compatibility has to be incorporated as follows;

7 $$\nabla \dot{u}=F\dot{\lambda }$$

where $\dot{\lambda }$ is the plastic multiplier that satisfies the complementarity conditions.

Therefore, the scaling applied with respect to the rate of work done by the reference tractions t as indicated above, in short, the scaling equation is denoted as follows;

8 $$\underset{{S}_{\sigma }}{\int }{t}^{T}\dot{u}dS=1$$

where $\dot{u}$ is taken as the exact velocity, *t* is traction vector.

And lastly, the complementarity conditions of the model are therefore incorporated as follows:

9 $${S}^{T}\dot{\lambda }=0, S\ge 0, \dot{\lambda }\ge 0$$

Nevertheless, a detailed description of the model in terms of lower bound principle, upper bound principles, bounds are documented below.

### Lower bound principle

In terms of the lower bound principles, the principle ensures that the strain-softening aligns with the governing equation of the limit analysis of the Finite element. However, the kinematic quantities, which are absent from the governing equations, appear as Lagrange multipliers when solving the problem. The main strength of the lower bound principle is that it allows for a lower bound on the exact collapse multiplier to be computed, through constructing a stress field that satisfies the constraints without necessarily being optimal^38^.

Maximize $$\alpha $$

10 $$ \begin{gathered}   {\text{Subjected to }}\nabla ^{T} \sigma  + b = 0{\text{, in V}} \hfill \\   {\text{P}}^{T} \sigma  = \alpha t{\text{on}}S_{\sigma }  \hfill \\   F^{T} \sigma  - k + s = 0 \hfill \\  \end{gathered}  $$

### Upper bound principle

As already stated that the problem at hand incorporates the kinematic quantities which the LEMs do not have the ability, therefore the upper bound is therefore intended to incorporate the compatible velocity field that satisfies the flow rule. A similar flow rule cannot be activated using the LEMs therefore this upper bound is indeed critical. In order to achieve that, the rate of work done by the reference tractions is scaled to unity and the objective function, which comprises the internal rate of work minus the contribution from the constant body forces, is then the collapse multiplier sought. The upper bound principles are denoted in Eq. (23).

Minimize $${\int }_{V}^{\cdot }{k}^{T}{\lambda }^{.}dV-{\int }_{V}^{\cdot }{b}^{T}{u}^{.}dV$$

11 $$ \begin{gathered}   {\text{Subjected}}\;{\text{to}}\;\nabla \dot{u} = F\mathop {\lambda ,}\limits^{.} \quad \lambda  \ge 0 \hfill \\   \mathop \smallint \limits_{{S_{\sigma } }} t^{T} \dot{u}dS = 1 \hfill \\  \end{gathered}  $$

### Bounds

The bounds are a critical part of the numerical simulation because this stage is used to verify the lower and upper bound principles to ensure that the bounds furnish with a collapse multiplier. Therefore the stress field is considered first, to ensure that they satisfy the yield condition and the equilibrium conditions as well as the boundary conditions. Such expression on how the bound is performed is documented by Eq. (12) (more details on the formulations see theories on Optimum 2G).

12 $$ \begin{aligned}   \alpha _{{ab}}  &  = \smallint _{V}^{.} k\lambda _{b} dV - \smallint _{V}^{.} b^{T} u^{.} dV \\     &  = \smallint _{V}^{.} (F^{T} \sigma _{b} )^{T} \lambda _{b}^{.} dV - \smallint _{V}^{.} b^{T} u^{.} dV \\     &  = \smallint _{V}^{.} \sigma _{b}^{T} F\lambda _{b}  - \smallint _{V}^{.} b^{T} u^{.} dV \\  \end{aligned}  $$

### Optum G2 computer code procedures

The model procedure followed in Optum G2 is readily available on the Optum Computational Engineering website under Optum G2^38^ analysis and examples. In summary, the procedure followed includes the project definition (Project File dialog, slope parameters dialog, Model layout), building the model in terms of model layers, input properties of the material, and model setting. After processing the provided information in the model, the results of the model are then presented in terms of SRF, gravity multiplier though the current study is interested in total displacement and shear dissipation, the material properties of the case are given in Table 1. The dimensions used for the slope are documented in Table 2.

**Table 2:** Slope dimensions used for the model.

Dimensions of the model		
Rise (m)	10	
Depth (m)	4	
Left (m)	10	
Run (m)	20	
Right (m)	15	
Slope degree	27°	

In terms of mesh size, it is well established that the element type, the mesh discretization and convergence toleration have a significant impact of the estimated slope failure^46,47^. Yet many scholars such as Ghavidel et al.^46^ and Lu et al.^47^ indicated that mesh density with fine mesh is the most accurate on and in this study a fine mesh was used in this study.

The shear strength properties of the fault are critical though the fault had the same rock properties with the country rock because it has no infill material. In this regard both cohesion and friction angle were considered and the details of the two shear strength properties of the rock are documented in Table 1.

## Results and discussion

A finite element method (FEM) called optimum 2G was utilized to establish the failure evolution process and shear dissipation of a faulted road-cut slope, the extreme rainfall of the study area was incorporated (which about 1000 mm/h). Detailed results of the simulation are documented in “Failure evolution process of the road cut slope” and “Shear dissipation of the material” below.

### Failure evolution process of the road cut slope

**Figure 5:**
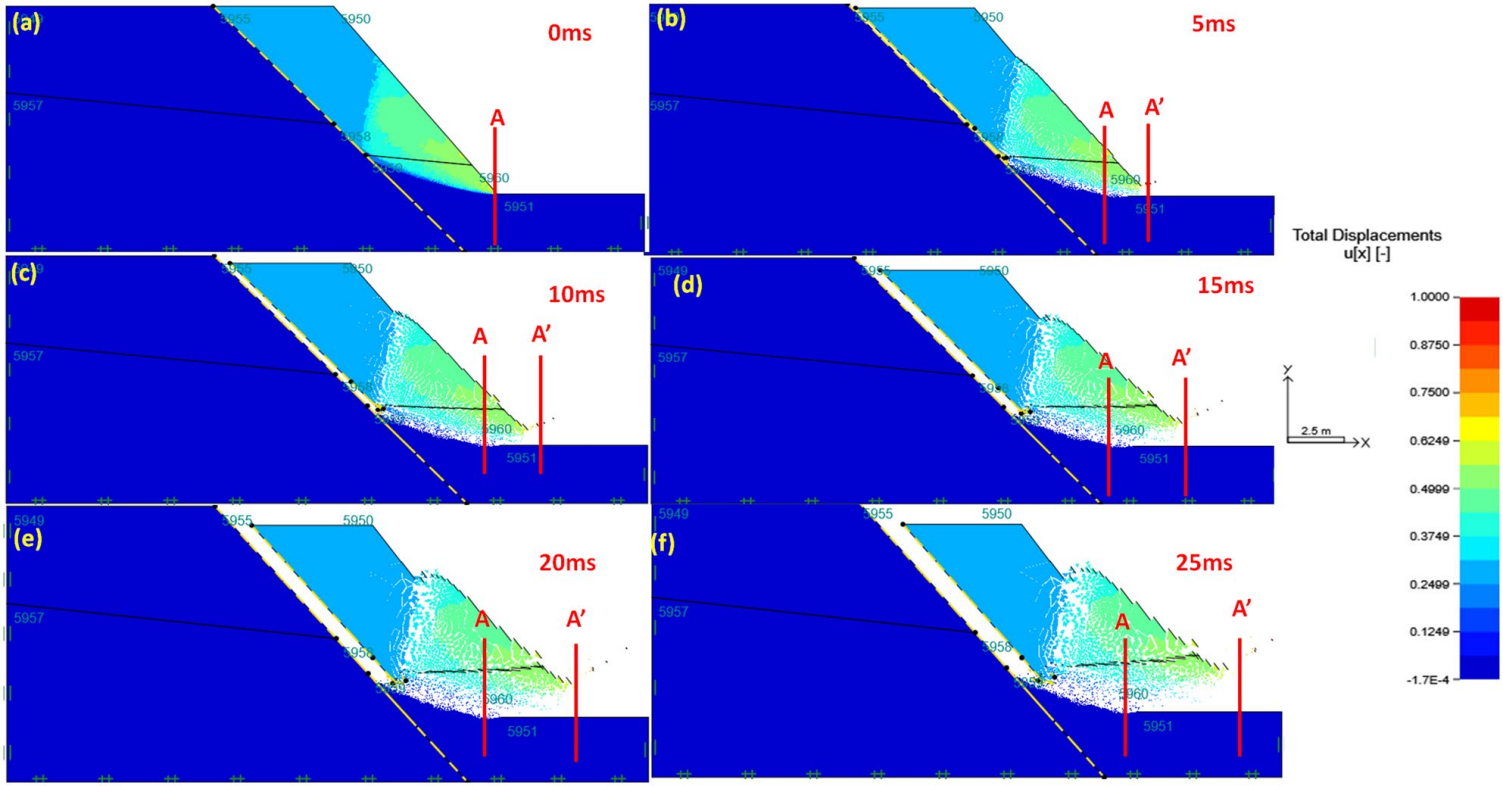
The total displacement of the slope in flow evolution process at (**a**) 0 ms; (**b**) 5 ms; (**c**) 10 ms; (**d**) 15 ms; (**e**) 20 ms; (**f**) 25 ms.

The results of the failure evolution are presented from Fig. 5a–f with the distance of material/soil/tailing movement measured in meters. Furthermore, the results are presented at a relatively short time interval of approximately 5 s per single computational cycle and it is assumed that the computational cycle is equal to the real lifetime. In Fig. 5a, it has been observed that when the computational simulation is at the rest of 0 s there is no movement of material at all, after an elapsed time of 5 s, the sliding mass of solid material (soil and rocks) has been observed (see Fig. 5b). The initial sliding of material has been denoted to begin at the toe part of the slope. In Fig. 5b, it was also noted that there was a severe deformation of material causing a total sliding distance of about 1.8 m, in short, this deformation occurred at about 0.36 m/s velocity. Indeed, one may deduce that the initiation of this sliding has taken at high velocity, which correlates with the dis-locking of the material, wherein several stresses (shearing, tension and compressive) interacted. Further simulations have shown that from t(time) = 10 to 25 s the sliding material were already reached the flat area. To be specific at t = 10 s, almost 10% of the sliding mas has reached the flat surface. Surprisingly, the velocity of the material was denoting to reduce as the duration of computational cycles increases (see Fig. 5c–f), these results correlate very well with some of the previous studies such as those of Zhang and his co-authors^30–36^. Although the mentioned previous authors were studying landslide flow-like structure the implementation merges with the present study. Therefore, one may argue that as material flows/slide the speed at which the material flows gradually reduced with distance.

Lastly, the run-out time of the simulation has shown that at about 25 s of the computational cycle the maximum distance in which the material to reach is approximately 5.2 m. Despite the fact that this is a numerical simulation, there has been a number of previous studies that conduct flow-like slides of landslides in steep terrain with similar properties as the present case studies and it has been documented that the distance in which the material run-out to is mostly influenced by the composition of the material as well as the smoothness of the ground flowing on. It is arguable that the numerical simulation presented in this section has some commonality with several previous studies which give the simulation potential to present realistic results. It is crucial to indicate that the computational simulation of the rate-independent model is based on the average process of sliding of the material and the real flow-sliding of solid mass slopes are similar to those of landslides. These simulations of flow-slide may govern by a complicated mechanism that may require a more sophisticated numerical tool with a high format of algorithms. In short, the presented model still present reasonable results of estimating the flow evolution of the slope and its run-out distance of final deposit, which are more critical for hazard assessment of the tailing slope stability.

### Shear dissipation of the material

The shear dissipation of the material is one of the critical concepts which integrates the turbulent kinetic energy and shear rate of material during flow-sliding of material. This fundamental principle allows verification of energy distribution relative to the resistance of the material.

**Figure 6:**
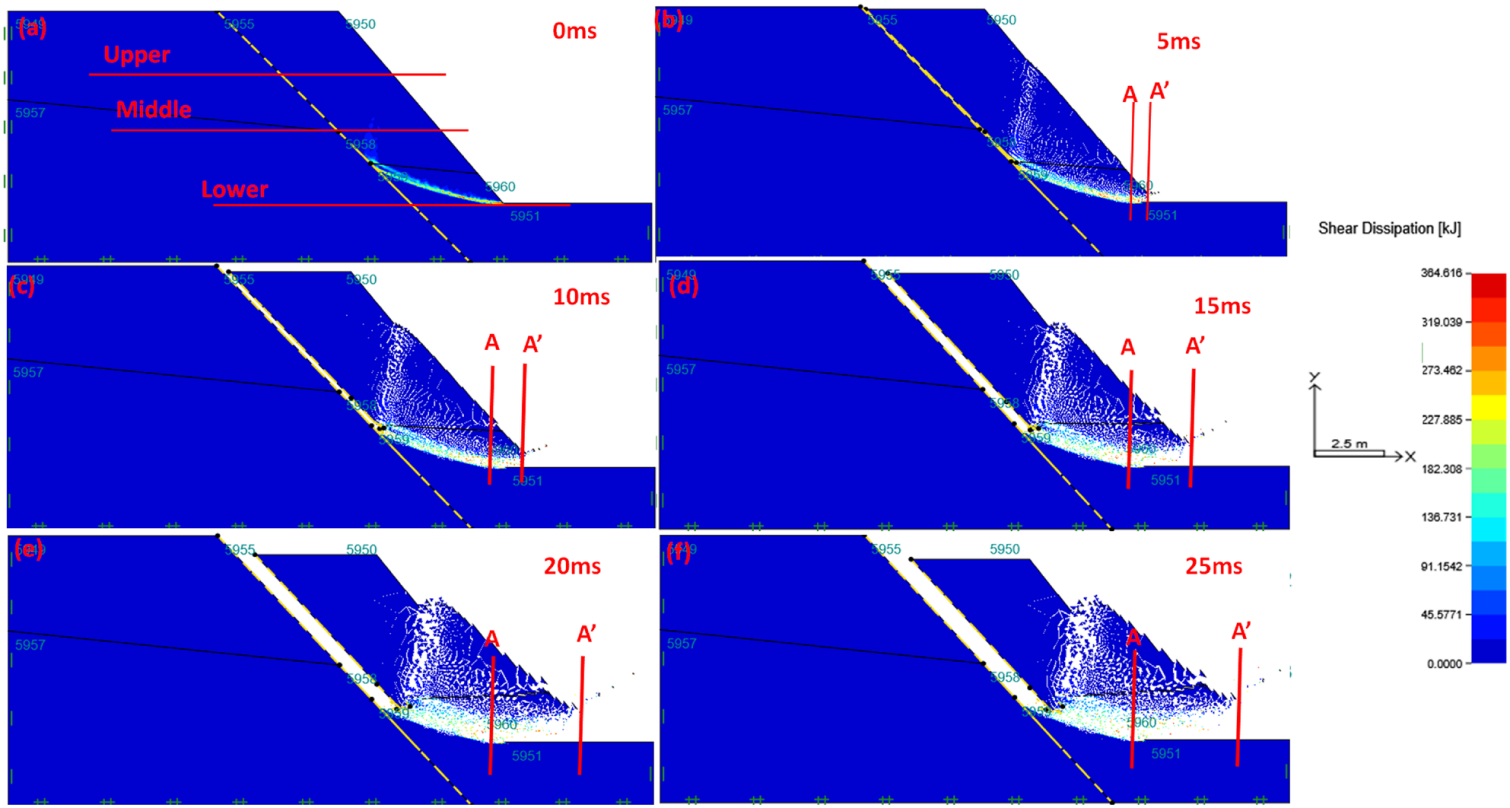
The shear dissipation of the slope in flow evolution process at (**a**) 0 ms; (**b**) 5 ms; (**c**) 10 ms; (**d**) 15 ms; (**e**) 20 ms; (**f**) 25 ms.

In this regard, the road slope was divided into three sections (upper, middle, and lower, (see Fig. 6a)) to compare the section regarding shear dissipation and its relation to flow sliding of the material. The results of the computational simulation in all cycles (from 0 to 25 s) (see Fig. 6a–f) have shown that the lower section of the slope experience extensive shear dissipation throughout the computational cycle. This result gives an impression that high kinetic energy and high shearing are expected at the toe of the slope during the initial stages of the evolution of flow until the last stage of evolution flow of material. Indeed, it makes sense that for the slope to collapse, the deformation starts at the bottom section of the slope, this process required more energy and more shearing of the material is expected. Yet minor deformation is expected of the material is expected to occur at the top part of the slope compared to the bottom part of the slope since the bottom part of the slope carries the slope weight and suffer with stresses.

Therefore, the previous section on failure evolution is well supported by the shear dissipation of the material. This analysis also presents some form of the accuracy of the model. Indeed, Zhang^30^ is of a view that if there is a correlation between shear dissipation and flow evolution of sliding material exposed to extreme rainfall fall, therefore, the computational simulation may present failure of a model rather than the failure of the slope.

It is crucial to indicate that the shear dissipation of the material appeared to correlate very well with the failure evolution of the slope simulated in “Failure evolution process of the road cut slope”. Nevertheless, the shear dissipation analysis is mostly conducted to understand the fundamental principles governed sliding looking into kinetic energy together with the shearing of material. This simulation also involves the resistance of the material with time. Indeed, from Fig. 6a–f, one may argue that the resistance of material increases with time as well as the distance. These observations were made wherein the speed of material gradually reduces with time. This implies that as material flow or slide, it phases changes gradually from liquid-like to solid-like, which gives the material stability to resist the flow.

## Concluding remarks

The purpose of this study was to present a numerical simulation that describes the failure evolution of a rock slope failure evolution, with a slope dominated with fault along a road cut. A case study of R71 road cut slope was used. The following conclusions are drawn from the study:

- It is well demonstrated that the present computational framework (optimum 2G) is capable of quantitatively reproducing the failure evolution process, the final run-out distance of the slope material.
- The simulation has evidenced that the flow evolution of material during extreme rainfall is expected to extend to the final deposit of 4.5 m, the simulation results correlates with field measurements and observations.
- The simulations also demonstrated that the distance in which material can reach (final run out) is largely controlled by the composition and phases the material undergone during flow evolution.
- The resistance of material has a major role in the run-out of the material; this resistance of the material is also controlled by shearing and absorbed kinetic energy during the process.
- The overall conclusion is that, for material to flow for a longer distance, high kinetic energy and more shearing of material are expected to take place during this process.

It is recommended that other sophisticated methods could be utilized to further the results such as establishing the failure mechanism and the kinematics of the slope instability.

## Data Availability

The data used to support the findings of this study are included in the article.
